# Co-localization of antibiotic resistance genes is widespread in the infant gut microbiome and associates with an immature gut microbial composition

**DOI:** 10.1186/s40168-024-01800-5

**Published:** 2024-05-10

**Authors:** Xuanji Li, Asker Brejnrod, Urvish Trivedi, Jakob Russel, Jonathan Thorsen, Shiraz A Shah, Gisle Alberg Vestergaard, Morten Arendt Rasmussen, Joseph Nesme, Hans Bisgaard, Jakob Stokholm, Søren Johannes Sørensen

**Affiliations:** 1 Section of Microbiology, Department of Biology, University of Copenhagen, 2100 Copenhagen, Denmark; 2 Section of Bioinformatics, Department of Health Technology, Technical University of Denmark, 2800 Kgs. Lyngby, Denmark; 3 COPSAC, Copenhagen Prospective Studies on Asthma in Childhood, Herlev and Gentofte Hospital, University of Copenhagen, Copenhagen, Denmark

**Keywords:** Gut microbiome, Infants, Metagenomics, Co-localization, Antibiotics resistance genes

## Abstract

**Background:**

In environmental bacteria, the selective advantage of antibiotic resistance genes (ARGs) can be increased through co-localization with genes such as other ARGs, biocide resistance genes, metal resistance genes, and virulence genes (VGs). The gut microbiome of infants has been shown to contain numerous ARGs, however, co-localization related to ARGs is unknown during early life despite frequent exposures to biocides and metals from an early age.

**Results:**

We conducted a comprehensive analysis of genetic co-localization of resistance genes in a cohort of 662 Danish children and examined the association between such co-localization and environmental factors as well as gut microbial maturation. Our study showed that co-localization of ARGs with other resistance and virulence genes is common in the early gut microbiome and is associated with gut bacteria that are indicative of low maturity. Statistical models showed that co-localization occurred mainly in the phylum Proteobacteria independent of high ARG content and contig length. We evaluated the stochasticity of co-localization occurrence using enrichment scores. The most common forms of co-localization involved tetracycline and fluoroquinolone resistance genes, and, on plasmids, co-localization predominantly occurred in the form of class 1 integrons. Antibiotic use caused a short-term increase in mobile ARGs, while non-mobile ARGs showed no significant change. Finally, we found that a high abundance of VGs was associated with low gut microbial maturity and that VGs showed even higher potential for mobility than ARGs.

**Conclusions:**

We found that the phenomenon of co-localization between ARGs and other resistance and VGs was prevalent in the gut at the beginning of life. It reveals the diversity that sustains antibiotic resistance and therefore indirectly emphasizes the need to apply caution in the use of antimicrobial agents in clinical practice, animal husbandry, and daily life to mitigate the escalation of resistance.

Video Abstract

**Supplementary Information:**

The online version contains supplementary material available at 10.1186/s40168-024-01800-5.

## Introduction

The first years of life are pivotal for the maturation of the gut microbiome [1, 2] and the healthy development of the host immune system [3]. Prolonged perturbation of the developing gut microbiome is linked to an increased risk for subsequent diseases, such as asthma [4] and allergies [5]. Together with the maturing gut microbiome, the antibiotic resistome—the pool of genes that contribute to antimicrobial resistance—also develops in the infant’s gut in the first few years of life. We recently showed that antibiotic resistance genes (ARGs) were enriched in the infant gut, mainly driven by the composition of *E. coli* [6].

Humans are not always exposed to antibiotics and ARGs in the gut may be maintained partly through a mechanism of “co-selection” between ARGs and other resistance genes. The physical linkage of multiple genes encoding different resistance phenotypes on the same genetic element (co-localization) is a widespread co-selection phenomenon [7] (Additional file 1: Figure S1). Through co-localization, selection for resistance against one antibacterial agent can result in the maintenance of resistance against other agents, termed “co-selection” [8]. Co-selection can favor a variety of ARGs in bacterial hosts that are more likely to be exposed to diverse co-selection agents and can further increase the persistence of certain bacteria and plasmids in the gut. Co-selection between ARGs and genes conferring resistance against agents such as antibacterial biocides and metals is frequently detected in the environment or animal gut [9–12]. However, the prevalence of this phenomenon in the human gut is largely unknown—especially in the immature gut, which is vital to understanding the spread and development of ARGs. This is a critical knowledge gap considering that humans are frequently exposed to co-selective agents in their daily lives such as biocides, which are usually utilized as antiseptics on the skin to prevent microbial infection [13], and metals, which are indispensable for humans.

In addition to resistance genes, bacteria have numerous other genetic tools to help them persist and thrive in their environments. In particular, virulence genes (VGs) play a critical role in determining bacterial pathogenicity and pose an important threat to human health [14]. Like ARGs, many VGs have been transferred among bacteria by horizontal gene transfer (HGT) [15, 16], which is one of the main ways in which human opportunistic pathogens acquire virulence in the course of evolution [17]. However, the early establishment of VGs in the gut microbiota remains unclear. It is possible that co-localization between VGs and ARGs may confer a selective advantage on VGs in bacteria that are likely to be exposed to antibiotics. To date, though, there have been no comprehensive investigations on the co-localization of VGs and ARGs in the infant gut microbiome. A better understanding of co-selection phenomena during early life could help elucidate the maintenance and proliferation of both ARGs and VGs, which is of key importance for efforts to alleviate the spread of ARGs and VGs.

To aim at a better understanding of co-selection phenomena in early life, we performed a comprehensive analysis of co-localization between ARGs and metal, biocide resistance genes and VGs, and characterized the common bacterial hosts in which this phenomenon occurs, through metagenomics sequencing of 662 fecal samples from healthy infants in the Copenhagen Prospective Studies on Asthma in Childhood 2010 (COPSAC_2010_) birth cohort (Table 1). We also evaluated the effects of antibiotic use on mobile ARGs and investigated the association between co-localization and microbial maturation of the infant’s gut.

**Table 1:** The cohort information in the study

Category	Variable	Statistics no. (%)
Child	Age, median (range)—years	1 (11 months–2)
	Sex (male)	341 (51.5)
	Race (Caucasian)	632 (95.5)
	Gestational age, median (range)—week	40 (29–42)
	Living area:	
	Rural	292 (46.5)
	Urban	336 (53.5)
	Birth Season:	
	Spring	177 (26.7)
	Summer	139 (21)
	Autumn	141 (21.3)
	Winter	205 (31)
	Delivery mode:	
	Vaginal	520 (78.5)
	Cesarean	142 (21.5)
	Siblings	382 (72.1)
	Type of home:	
	House	229 (42.4)
	Apartment	311 (57.6)
	Food:	
	Breastfeeding + solid food	98 (14.8)
	Solid food	562 (85.2)
	Breastfeeding history	653 (98.9)
	Total days of breastfeeding (mean ± sd)	250 ± 165
	Fish oil	327 (49.5)
	Antibiotics in the year prior to sampling	311 (47)
During pregnancy	Pet:	
	Cat	133 (20.2)
	Dog	123 (18.8)
	Antibiotics	271 (40.9)
	Smoking	47 (7.1)
	Alcohol	34 (5.1)
Maternal information	Age, median (range)—years	32 (19–48)
	Mother BMI—kg/m^2^ (mean ± sd)	23.6 ± 4.3
	Income level:	
	Low	63 (9.5)
	Medium	352 (53.3)
	High	246 (37.2)
	Education level:	
	Low	53 (8%)
	Medium	423 (63.9%)
	High	186 (28.1%)

Antibiotic information for infants refers to antibiotic use during 1^st^ year prior to sampling. Antibiotic information for pregnancy refers to antibiotics used during delivery

## Materials and methods

### Study population and sample collection

The study subjects were participants in the population-based COPSAC_2010_ mother-child cohort consisting of 700 mother-child pairs [18, 19]. The fecal samples investigated in this study were gathered from infants aged 11 months to 2 years (median age 1 year), either at the COPSAC research unit or by the parents at home, following instructions. The fecal samples were collected in sterile plastic containers and transported to Statens Serum Institut (Copenhagen, Denmark) with a median transport time of 2 days at ambient temperature. Each sample was mixed on arrival with 1 mL of 10% vol/vol glycerol broth (SSI, Copenhagen, Denmark) and frozen at – 80 °C until further.

### Covariates

At scheduled visits to COPSAC clinics, participants provided information regarding the use of antibiotics (including any treatment prior to sampling; summarized in Additional file 1: Figure S2), in addition to various demographic details such as age, sex, race, gestational age at delivery, siblings, living area, birth season, income, smoking, type of home, and pet ownership (Table 1). This information was cross-checked against registration records.

### Metagenomics sequencing and data processing for fecal samples

Bacterial DNA from 663 fecal samples was extracted using the PowerMag^®^ Soil DNA Isolation Kit optimized for the epMotion robotic platform model according to extraction instructions. The Kapa Hyper Prep kit (for Illumina) was used for sequencing library preparation. Fecal DNA samples were sequenced using the Illumina NovaSeq apparatus by Admera Health (USA). Out of the 663 samples, one sample failed to produce a library. To mitigate a batch effect, the 662 samples were processed in the same batch, including library preparation and DNA extraction. GNU Parallel v20180722 [20] was used for parallelized preprocessing during bioinformatics analysis. The adapter sequences were trimmed by BBDuk (BBTools v38.19) using the default setting except for the following parameters: “ktrim=r k=23 mink=11 hdist=1 hdist2=0 ptpe tbo”. Reads shorter than 50 bases and low-quality sequences were removed by Sickle v1.33 [21]. Human genome contaminants were filtered out using BBMap (BBTools v38.19) with the default setting. Short reads were assembled into contigs individually using SPAdes v3.12.0 under the default settings [22]. Metagenomic sequencing coverage was analyzed using Nonpareil v3.30 with kmer mode [23]. Taxonomic classification of microbial communities was inferred with MetaPhlAn v2.7.5 [24], which is embedded in the humann2 v0.11.2 pipeline [25]. The binning of metagenomics contigs into metagenomically assembled genomes (MAGs) individually was performed by three binners in the metaWRAP pipeline (v1.2.2) [26]: MetaBAT2 v2.12.1 [27], MaxBin2 v2.2.6 [28], and Concoct v1.0.0 [29]. The quality assessment of MAGs was carried out using CheckM v1.0.12 [30], and only MAGs with at least 90% integrity and no more than 5% contamination were retained. One sample did not generate any MAGs. In total, 452 dereplicated MAGs were generated for the 661 samples. GTDB-Tk toolkit (v1.7.0 and GTDB-Tk reference data r202) was used to infer the bacterial taxonomic assignments of MAGs [31]. Open reading frames (ORFs) in contigs were identified with Prodigal v2.6.3 in META mode [32].

### Identification of resistance genes, MGEs, virulence genes, and integrons

Resistance Gene Identifier [33] was used to search for ARGs within the predicted ORFs by aligning the amino acid sequences of ORFs to the Comprehensive Antibiotic Resistance Database (CARD v3.0.7). The significance cut-offs “Perfect” (100% identity and 100% reference sequence coverage) and “Strict” (a match higher than the bitscore of the curated BLASTP bitscore cutoff) were used as the thresholds for filtering ARGs. Diamond blastp was used to search for biocide resistance genes (BRGs) and metal resistance genes (MRGs) in the predicted ORFs by aligning the amino acid sequences of ORFs to sequences in a database of antibacterial biocide and metal resistance genes (BacMet v2.0) [34] using the more sensitive mode and k1 option [35]. Thresholds of 90% identity and 80% query coverage were used to predict BRGs and MRGs. Mobile genetic element (MGE) homologs were characterized using the PFAM [36] and TnpPred [37] databases through HMMSEARCH (v3.1b2)[10, 38], with “-cut_ga” as the threshold. If ORFs had multiple hits, we only kept the one with the lowest E-value. Diamond blastp search was also performed to predict the families of bacterial virulence factors from the amino acid sequences of the predicted ORF using the VFDB (Virulence Factor Database) (updated version on Sep 10, 2021) [39] with the more sensitive mode and k1 option [35]. Thresholds of 90% identity and 80% query coverage were used to predict VGs. *AttC* recombination sites, promoters, and *attI* sites for the integrons were identified by IntegronFinder with the default setting [40]. The distribution of resistance genes and VGs in bacterial species is shown in Additional file 1: Figure S3. The profiles of resistance genes and VGs are shown in Additional file 1: Figure S4. The choice of different gene thresholds using Diamond blastp was based primarily on different databases and the need to balance specificity and sensitivity. For CARD, the recommended “Perfect” and “Strict” cutoffs were used for stringent characterization, whereas for BacMet and VFDB, they were used to capture a wider range of genetic determinants.

### Calculation of gene abundance

The alignment of clean reads against the ORFs was performed by the Bowtie2 aligner [41]. Samtools idxstats [42] was used to calculate the number of mapped reads in the bam file. We used values of gene coverage per million (GCPM) for ORFs to normalize the length of ORFs and sequencing depth [10]. Because the sum of GCPM values of all the ORFs in each sample is the same, the abundance of ORFs is thus comparable between samples. The formula to calculate GCPM is $\frac{\text{(counts /gene length)} \times 10^{6}}{\sum\nolimits^{\text{n}}_{1} \text{counts /gene length}}$, where counts represent the number of mapped reads, gene length represents the ORF length, and n represents the total number of predicted ORFs in each sample.

### Identification of bacterial origin of genes

The bacterial species from which gene-containing chromosomal contigs originated were traced from the taxonomic classification of MAGs. In this way, we were able to classify genes according to their bacterial species of origin.

### Co-localization analysis and stochasticity analysis of co-localization

Co-localization represents the physical relationship between genes on the same assembly contig. We selected representative plasmid contigs to demonstrate class 1 integrons; first, we deduplicated co-localization contigs based on gene combinations, then removed the co-localization contigs whose gene combinations were included in other co-localization contigs and the representation contigs were finally obtained. Gene arrangements in the representative contigs were visualized using gggenes (https://wilkox.org/gggenes/). In this study, we refer to co-localization associated with MGEs occurring on plasmids as mobile co-localization. The three types of mobile co-localization phenomena investigated here are (1) ARGs, BRGs, and MGEs; (2) ARGs and MGEs; and (3) VGs and MGEs. The ARGs and VGs in categories 2 and 3 are also referred to as mobile ARGs and mobile VGs.

In addition, we assessed the stochasticity of co-localization using an enrichment score, which we defined as the fold difference between the actual and the expected numbers of co-localized contigs. This was calculated as $\frac{\mathrm{actual\;number\;of\;co}-\mathrm{localization\;contigs}}{\mathrm{expected\;number\;of\;co}-\mathrm{localization\;contigs}}$ . A score higher than 1 served as evidence of enrichment in the gut. In which, the expected number of co-localization contigs was calculated as $\mathrm{the\;total\;number\;of\;contigs}*\mathrm{The\;expected\;probability}\;p$ (shown below). A binomial test with FDR adjustment [43] (R function “binom.test”) was used to test whether the actual number of co-localization contigs was significantly higher than the expected number of co-localization contigs (*p* < 0.001 as significance cutoff), i.e. whether an enrichment score was statistically significant and a given pattern of gene co-localization occur by chance. The x, n, and p in the R function “binom.test” represent the actual number of co-localization contigs carrying resistance genes for drugs A and B, the total number of contigs, and the expected probability of contigs carrying resistance genes for drugs A and B, respectively. The expected probability p (the probability of two genes being located in the same contigs) is calculated as $\left(\frac{\mathrm{the\;number\;of\;contigs\;carrying\;drug\;A\;resistance}}{\mathrm{the\;total number\;of\;contigs}}\right)\times \left(\frac{\mathrm{the\;number\;of\;contigs\;carrying\;drug\;B\;resistance}}{\mathrm{the\;total\;number\;of\;contigs}}\right)$.

### Source identification of co-localization contigs

We used PPR-Meta v1.1 [44] to classify metagenomics sequences as coming from chromosomes, plasmids, or phages. As the software suggested, a probability score of 0.7 (between 0 and 1) was used as the threshold. The bacterial species of origin of chromosomal contigs was traced from the taxonomic classification of MAGs.

### Statistical analysis

The statistical software “R” was utilized for data organization and statistical analyses [45].

### Modeling analysis of co-localization between ARGs and BRGs among different bacteria

To check for differential patterns of co-localization between ARGs and BRGs in different bacteria and adjust for the different lengths of co-localized contigs, we built a generalized linear model with a quasipoisson distribution using R function “glm”, to evaluate the association between the numbers of co-localized ARGs (dependent variable), the presence of BRGs, and the log-transformed length of co-localized contigs in the four main bacterial phyla. In this model, the number of co-localized ARGs is modeled as a function of the presence of BRGs with an offset by the logarithm of contig length, under the assumption of a quasipoisson distribution with a log link function.

### Modeling analysis of mobile ARGs and virulence genes in plasmid contigs

We built a logistic regression with a binomial distribution using R function “glm” and used a binomial regression to explore the association between the presence of mobile VGs or ARGs (i.e., VGs or ARGs with MGEs) in contigs (dependent variable), the presence of these contigs in plasmids, and the log-transformed length of these contigs. This model represents a logistic regression model where the binary response variable the presence of mobile VGs or ARGs is modeled as a function of the predictor variables the presence of these contigs in plasmids and the logarithmically transformed length of contigs, under the assumption of a binomial distribution.

### Correlations between gut microbial maturity and the abundance of co-localized ARGs

In previous work, we demonstrated how to calculate a microbiota-by-age z-score (MAZ) for evaluating gut microbiome maturity across ages and asthma risk at age 5, respectively [2]. Here, we created a linear model using the R function “lm” to explore the linear association between MAZ scores at 1 year of age generated from the previous work (as the response variable) and the abundance of *E. coli* or co-localized ARGs (as dependent variables). This analysis aims to understand to what extent MAZ scores are affected by *E. coli* or co-localization ARG abundance.

## Results

### Co-localization in the infant gut was most common between tetracycline or fluoroquinolone ARGs and other ARGs

**Fig. 1:**
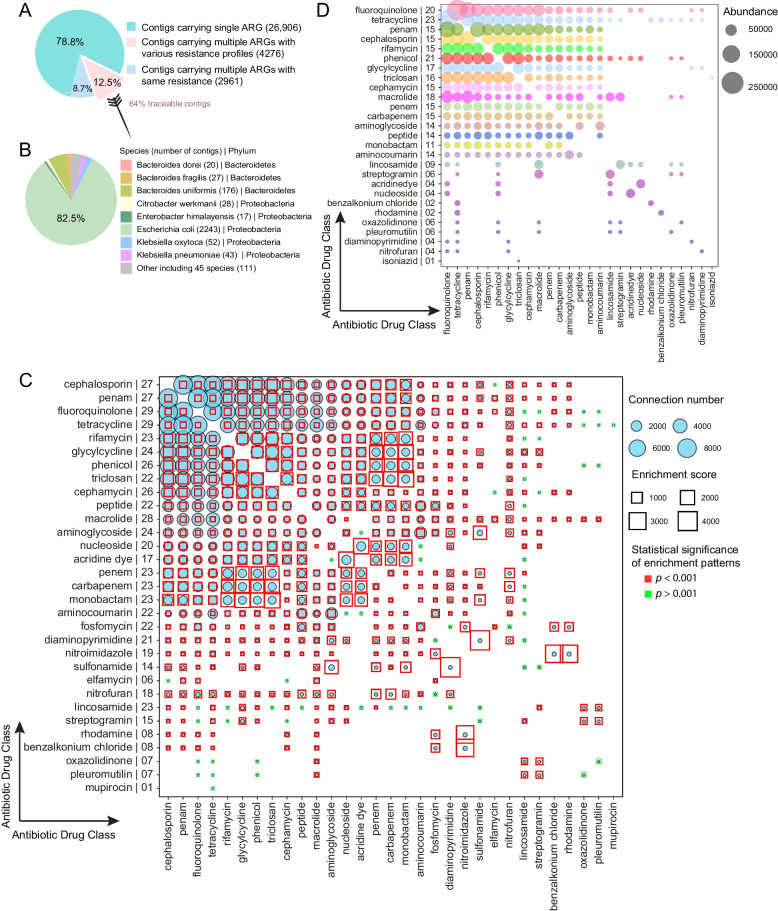
Overview of co-selection between ARGs in the infant gut. **A** Proportion of contigs carrying different numbers of ARGs. **B** Taxonomic origin of contigs carrying multiple ARGs with different resistance profiles. **C** Co-localization bubble chart representing the drug classes related to different ARGs. The number of connections between ARGs targeting different drug classes in the contigs, and the associated enrichment scores, are shown in the figure. On the *y*-axis, the number to the right of the name indicates the number of other drug classes represented in the co-localization arrangements. The size of the bubble indicates the number of connections in the contigs. The enrichment scores higher than 1 are indicative of enrichment in that co-localization arrangement. A binomial test with FDR adjustment was used to test the statistical significance of enrichment patterns (*p* < 0.001 was set as the significance cutoff; red square frame represents *p* < 0.001 and blue square frame represents *p* > 0.001). A significant *p* value indicates that the occurrence of that specific pattern of gene co-localization would not be expected by chance. The size of the square frame represents the magnitude of the enrichment score. **D** The bubble chart represents the drug classes targeted by 167 MDR ARGs. The size of the bubble is proportional to the abundance of MDR ARGs potentially conferred resistance to two drug classes. On the *y*-axis, the number to the right of the name indicates the number of other drug classes represented in the co-localization arrangements.

We conducted a comprehensive investigation into the co-selection of ARGs, with a focus on two phenomena: (1) co-resistance (multiple ARGs conferring resistance to different drugs that are all located in the same genetic element, and (2) MDR ARGs (multidrug resistant ARGs). Of all the contigs that were found to contain ARGs, 21.2% carried multiple ARGs and more than half of these contained ARGs known to confer resistance against different drugs (Fig. 1A). Using metagenomics assembled genomes (MAGs), we traced the contigs carrying multiple ARGs with different resistance profiles to 55 bacterial species of origin, representing 5 phyla. The majority of the traceable contigs were from Proteobacteria, particularly *E. coli* (Fig. 1B).

To reflect gene co-resistance more intuitively, we transformed the co-resistance network of 4276 ARG-carrying contigs into a network that depicts resistance against 31 classes of drugs. We collapsed together all genes conferring resistance to a given drug class based on the CARD database and examined how often a resistance gene for one class was found to be co-localized with a resistance gene for another class (Fig. 1C). Genes potentially conferring resistance to cephalosporin, penam, fluoroquinolone, and tetracycline were the most likely to be co-localized with other drug resistance genes. Co-localization with fluoroquinolone and tetracycline resistance genes was most common, followed by macrolides, cephalosporin, and penam.

Instead of setting the threshold of contig length, we used enrichment scores from a broader perspective to assess the stochasticity of co-localization in terms of antibiotic drugs to which genes can potentially convey resistance. Among different types of ARGs, enrichment scores for co-localization ranged from 1.65 to 4126 (mean (SD): 365(554), Fig. 1C). With the exception of ARGs related to lincosamide, most of the co-localization between ARGs was significantly enriched compared to the expected values (binomial test; adjusted *p* < 0.001), suggesting that the co-localization was not the result of chance.

Among the 409 antibiotic-resistance genes detected in the infant’s gut, 167 potentially provided resistance against at least two antibiotics. These MDR ARGs potentially conferred resistance against 27 drug classes in total. Of these, multiple resistances to tetracycline, phenicol, and fluoroquinolone were most prevalent (Fig. 1D). Indeed, the most abundant MDR ARGs in the infant gut were those that potentially conferred resistance against both tetracycline and fluoroquinolone (Fig. 1D).

### Co-localization between ARGs and biocide or metal resistance genes is frequently detected in the infant gut, especially in E. coli

**Fig. 2:**
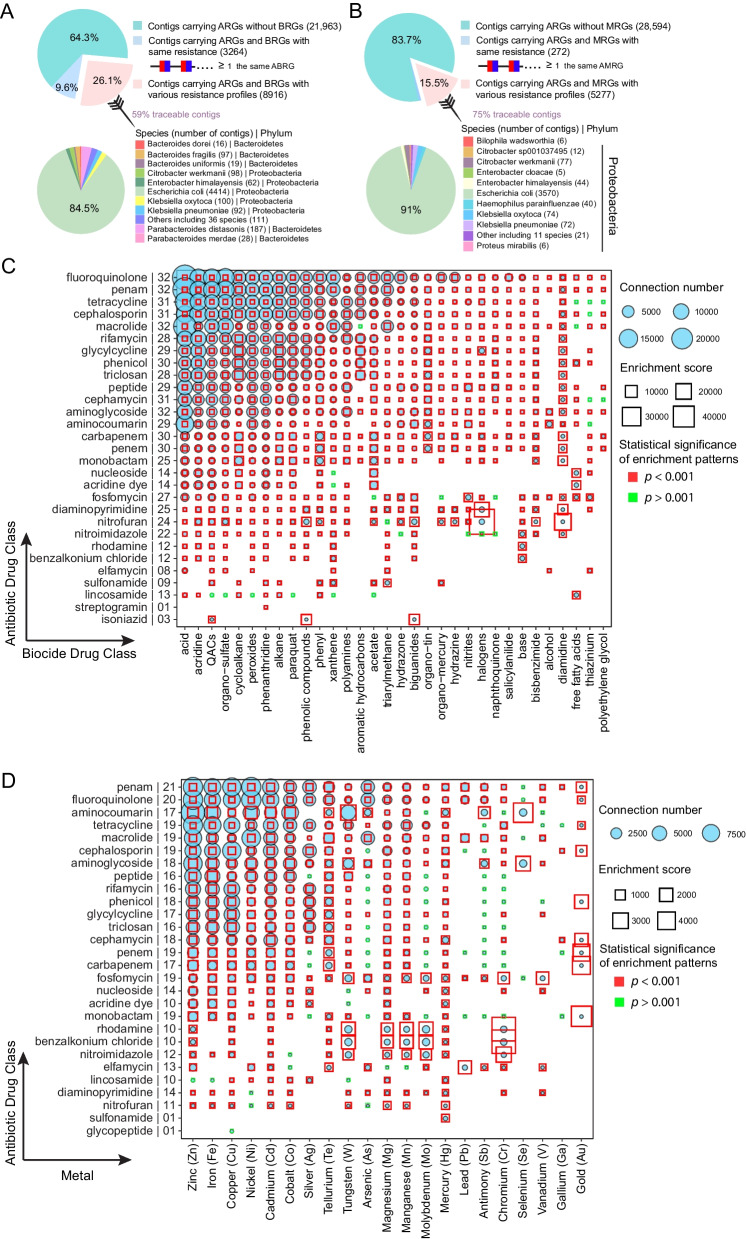
Co-localization between ARGs and BRGs or MRGs was frequently detected in the infant gut, especially in *E. coli*. **A** Proportion of contigs carrying ARGs and BRGs, and the taxonomic origin (bacterial species and phylum) of contigs carrying ARGs and BRGs with different resistance profiles. The red and blue modules on one ABRG gene graphically symbolize antibiotic and biocide resistances, respectively. **B** Proportion of contigs carrying ARGs and MRGs, and taxonomic origin (bacterial species and phylum) of contigs carrying ARGs and MRGs with different resistance profiles. The red and blue modules on one ABRG gene graphically symbolize antibiotic and biocide resistances, respectively. **C**, **D** Co-localization bubble charts representing drug classes targeted by ARGs and BRGs with different resistance profiles (**C**) and between drug classes targeted by ARGs and metals targeted by MRGs (**D**). The number of connections in the contigs between ARGs and BRGs/MRGs with different targets, along with the enrichment scores, are shown in the figure. On the *y*-axis, the number to the right of the antibiotic drug class indicates the number of biocide drug classes (**C**) or metals (**D**) represented in the co-localization arrangement**.** The size of a bubble indicates the number of connections in the contigs. A binomial test with FDR adjustment was used to test the statistical significance of enrichment patterns (*p* < 0.001 was set as significance cutoff; red square frame represents *p* < 0.001 and blue square frame represents *p* > 0.001). A significant *p* value indicates that the occurrence of that specific pattern of gene co-localization would not be expected by chance. The size of the square frame indicates the magnitude of the enrichment score.

We investigated in detail the co-localization between different types of resistance genes in the early gut, with respect to both co-resistance and MDR genes (a single gene conferring resistance to both antibiotics and biocides, ABRGs). Among all of the contigs that contained ARGs, 26.1% also carried BRGs that targeted a different mode of resistance (Fig. 2A). When we used MAGs to trace the bacterial origin of these contigs, we found that they originated from 5 phyla and 47 species; 92.2% of the traceable contigs carrying ARGs and BRGs with different resistance profiles came from Proteobacteria, of which 84.5% originated from *E. coli* (Fig. 2A). In the same way, we found that 15.5% of contigs with ARGs also carried MRGs (Fig. 2B), and 99.5% of the traceable co-localized contigs were of Proteobacterial origin, 91% from *E. coli* (Fig. 2B).

To better visualize gene co-resistance, we transformed the co-resistance network between ARGs and BRGs into a network representing 29 antibiotic drug classes and 32 biocide drug classes (Fig. 2C). The ARGs that most frequently co-occurred with BRGs were those that targeted fluoroquinolone, penam, tetracycline, cephalosporin, and macrolides. Moreover, ARGs associated with resistance to fluoroquinolone, penam, macrolides, and aminoglycoside were found to be co-localized with BRGs associated with all 32 biocides. The co-resistance network between ARGs and MRGs involved 28 antibiotic drug classes and 21 metals (Fig. 2D). The ARGs that most frequently co-occurred with MRGs were those potentially conferring resistance to penam, fluoroquinolone, aminocoumarin, tetracycline, and macrolides; genes for penam resistance were found to be co-localized with resistance genes associated with all 21 metals. The co-localization enrichment scores were between 1.65 and 40,580 for combinations of ARGs and BRGs (mean (SD) 537(1814)) (Fig. 2C) and between 0.47 and 4,651 for ARGs and MRGs (mean (SD): 236(473)) (Fig. 2D). With the exception of ARGs related to lincosamide, most of the co-localization between ARGs and BRGs was significantly enriched compared to expected values (binomial test; adjusted *p* < 0.001, Fig. 2C), while 15% of the co-localization between ARGs and MRGs could have occurred by chance (binomial test; adjusted *p* > 0.001, Fig. 2D). With respect to multiple resistances, approximately 10% of ARGs in the infant gut also conferred resistance to biocides. The antibiotic and biocide resistance profiles that were most commonly implicated in multiple resistances were those related to fluoroquinolone and quaternary ammonium compounds (QACs), respectively (Additional file 1: Figure S5).

### Virulence genes are associated with an immature gut microbiome

**Fig. 3:**
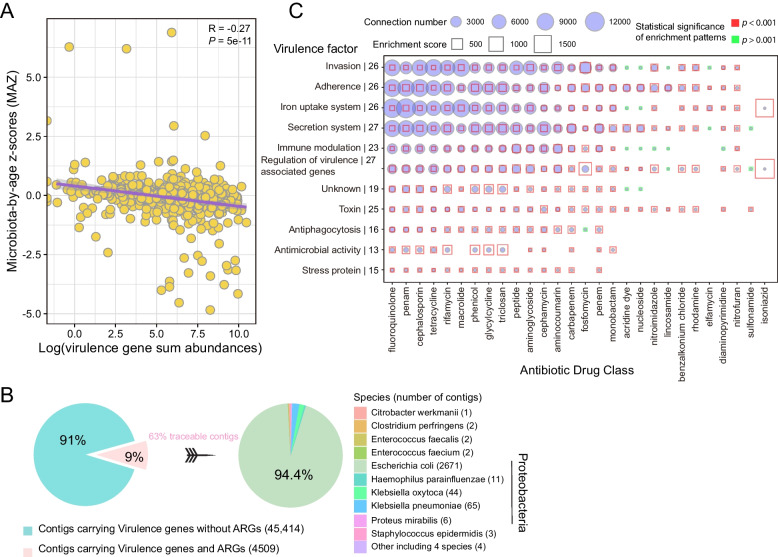
The association between VGs and gut microbial maturation, and co-localization between ARGs and VGs. **A** The association between total abundance (log-transformed) of VGs and MAZ score. The confidence interval for the slope of the linear regression line is plotted as an illustration; the inference was performed with the Spearman correlation coefficient R and the corresponding *p*-value (*p* < 0.05 as significance cutoff). **B** Proportion of contigs carrying ARGs and VGs, and taxonomic origin (bacterial species and phylum) of contigs carrying both ARGs and VGs. **C** Co-localization bubble chart depicting drug classes associated with ARGs and virulence factors encoded by VGs. The number of connections in the contigs between ARGs representing different drug classes and genes encoding different virulence factors is shown in the figure, along with enrichment scores. On the *y*-axis, the number to the right of each virulence factor indicates the number of antibiotic drug classes represented in the co-localization arrangements. The size of the bubble indicates the number of connections in the contigs. A binomial test with FDR adjustment was used to test the statistical significance of enrichment patterns (*p* < 0.001 was set as the significance cutoff; red square frame represents *p* < 0.001 and the blue square frame represents *p* > 0.001). A significant *p*-value indicates that the occurrence of that specific pattern of gene co-localization would not be expected by chance. The size of the square frame indicates the magnitude of the enrichment score.

The establishment and development of VGs in the human gut is still unknown. Here we assessed the association between the abundance of VGs and gut microbial maturity at 1 year (Fig. 3A). In our previous study [2], we obtained ‘microbiota by age’ *z*-score (MAZ) for each infant by training 16S datasets at different time points by machine learning. This allowed us to assess gut microbial maturity, with higher MAZ values representing higher maturity, as shown in our previous paper [2]. In this study, we utilized MAZ scores at 1 year of age derived from our previous study. Spearman correlation analysis revealed that a higher load of VGs was significantly correlated with lower MAZ scores (Fig. 3A, R = − 0.27, *p* < 0.001), indicating that infants with a high load of VGs had more immature gut microbiomes.

We investigated the co-localization of ARGs and VGs in bacterial contigs in the infant gut; 9% of contigs with VGs also carried ARGs, with 94% of the traceable co-localized contigs originating from *E. coli* (Fig. 3B). The co-localization network of ARGs and VGs involved 28 drug classes and 11 virulence factors (Fig. 3C). Of particular interest were genes associated with virulence gene regulation and secretion systems, which were found to be co-localized with ARGs representing 27 different classes of antibiotics. Similarly, genes associated with invasion, adhesion, and iron uptake systems, also frequently appeared on the same contigs as ARGs. Enrichment scores for co-localization arrangements ranged from 0.98 to 1,943 (mean (SD): 97(177)) (Fig. 3C), and most of this enrichment was considered significant (binomial test; adjusted *p* < 0.001, Fig. 3C).

### The high frequency of co-localization in Proteobacteria is independent of high ARG content and contig length

**Fig. 4:**
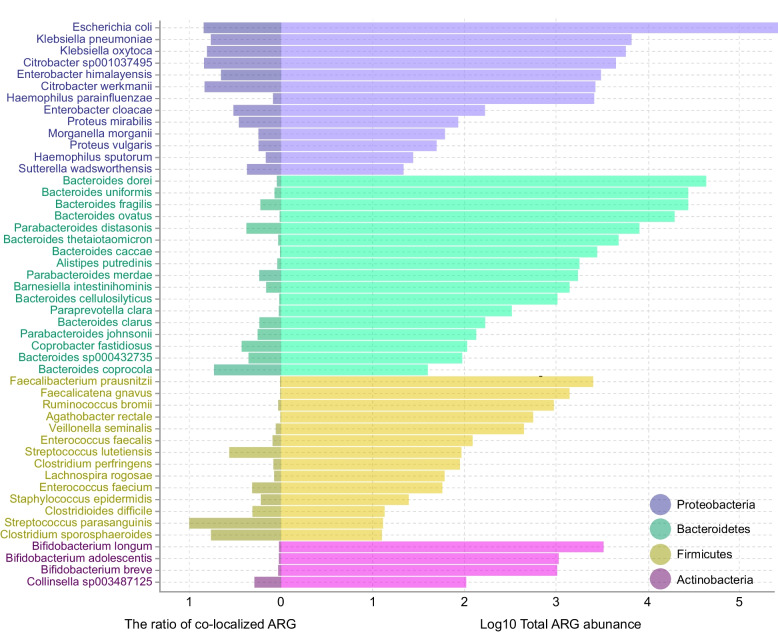
The log-transformed total abundance of ARGs and the ratio of co-localized ARGs with BRGs in different bacterial species

Overall, we found that co-localization was more likely to occur in genomes in phylum Proteobacteria*,* especially in *E. coli*. The potential explanation for a high frequency of co-localization could simply be high ARG content (both including copy number and the number of contigs) or variance in contig length. To test this hypothesis, we investigated patterns of co-localization between ARGs and BRGs (Fig. 4).

In the 50 bacterial species examined, we did not detect a significant positive correlation between the ratio of co-localized ARGs and total ARG abundance (Pearson correlation; *R* = − 0.091, *p =* 0.53). This suggests that there may beno uniform pattern of co-localization between ARGs and BRGs in all bacteria; instead, it appears that patterns of co-localization are specific to individual taxa. As shown in Fig. 4, despite the large numbers of ARGs present in most species of Bacteroidetes*,* Firmicutes, and Actinobacteria, only a small number of these were found to be co-localized with BRGs. To further verify this disproportionate pattern of representation and exclude any confounding effect of possible differences in the lengths of assembled contigs, we built a generalized linear model with a quasipoisson distribution to explore the association between the number of ARGs (the dependent variable), the presence of BRGs, and the log-transformed length of contigs in the four main phyla. It turned out that Proteobacteria explained ≈ 29% of the variance in the co-localization of ARGs and BRGs (Additional file 1: Figure S6), suggesting that this phylum was indeed the most important source for the co-localization of ARGs and BRGs.

### Antibiotics cause short-term abundance change in mobile ARGs; virulence genes exhibit higher potential for mobility than ARGs

Resistance and VGs can be widely transferred horizontally between bacteria via plasmids. We next explored mobile co-localization phenomena on plasmids. The gene elements carried on plasmids and co-localization profiles are listed in Additional file 1: Figure S7.

Within the infant gut, class 1 integrons were the most common co-localization genetic elements residing on plasmids (Additional file 1: Figure S8). We detected 36 representative plasmid contigs carrying ARGs, BRGs, and MGEs. Of these, 22 contained a complete class 1 integron, featuring a 3′-conserved segment containing a sulphonamide resistance gene (*sul*) and a QAC resistance gene (*qac*), a 5′-conserved segment carrying an integrase, and a gene cassette. In total we found that 17 ARGs were integrated into the cassette, including six aminoglycoside resistance genes (*aadA/2/3/5/6/8b*), seven diaminopyrimidine resistance genes (*dfrA1/5/7/12/15/16/17*), two sulfadiazine resistance genes (*sul1/3*), one beta-lactamase resistance gene (*CARB-3*), and one phenicol resistance gene (*cmlA1*).

**Fig. 5:**
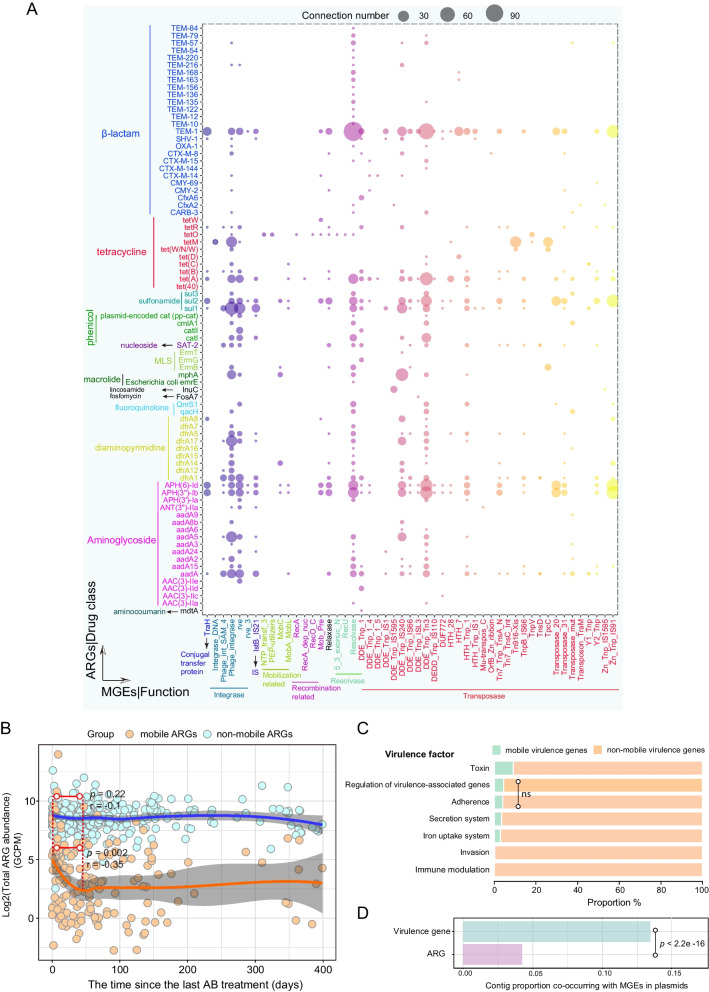
Co-localization of ARGs, BRGs/VGs, and MGEs on plasmids in the infant gut. **A** Co-localization bubble chart representing ARGs and MGEs in plasmid contigs. The size of the bubble is proportional to the number of connections in the contigs. **B** The effects of antibiotic treatment on log-transformed total abundance of mobile ARGs and non-mobile ARGs with time. The confidence interval for the slope of the linear regression line is shown. Orange and blue dots represent the 311 infants who received antibiotics in the first year. Infants lacking detectable mobile ARGs are not represented in the figure. The *X* coordinate represents the time of the most recent antibiotic treatment prior to sampling for each infant. The significance level and Pearson correlation coefficient R are shown in the figure; dotted lines denote the window in which the Pearson correlation was calculated (from 0 to 45 days). AB represents antibiotics. **C** The relative proportions of mobile and non-mobile VGs related to seven virulence factors. Pairwise Fisher's exact test was carried out for the comparison of the relative proportions of mobile and non-mobile genes within each of the seven groups. Multiple comparisons were adjusted for FDR. Except for the pairwise comparison of adherence- and regulation-associated genes (*p* = 0.06, indicated by ns), there were significant differences between all paired comparisons (*p* < 0.001).** D** The proportion of all contigs with ARGs/VGs and MGEs that were found in plasmids. *p* value from Fisher’s exact test is shown in the figure

We identified 80 mobile ARGs that co-localized with 51 MGEs on plasmids (Fig. 5A). Of the mobile ARGs, 26 potentially encoded resistance to β-lactams, 17 to aminoglycoside, and 10 to tetracycline. Among the different MGEs, transposases, and integrases were most commonly found beside ARGs on plasmids. When we examined the influence of antibiotic use on the abundance of mobile ARGs in the infant gut, we found a transient effect: in infants who received antibiotics, the total abundance of mobile ARGs in the gut peaked on the first day after treatment (Fig. 5B), significantly declined over the next ca. 45 days (Pearson correlation, *R* = − 0.35, *p =* 0.002), and finally reached a stable plateau close to the levels found in the guts of infants who had not received any antibiotic treatment. In contrast, putatively non-mobile ARGs, i.e., those not on the same contigs as MGEs, did not exhibit any change in abundance in the 45 days after antibiotic usage (*R* = − 0.1, *p =* 0.22). These results also indicate that antibiotic treatment exerted an influence mainly on mobile ARGs.

Among 2128 VGs detected, 321 of them were mobile (Fig. 5C and Additional file 2: Table S1) in which genes associated with toxins showed the strongest mobility. We detected significant differences in the proportions of mobile genes among virulence categories (pairwise Fisher's test with FDR adjustment, *p* < 0.001 for each comparison excluding genes related to adhesion and regulation, Fig. 5C). In particular, we observed that a significantly higher proportion of plasmid contigs carrying VGs than of the plasmid contigs carrying ARGs (Fisher’s exact test, *p* < 0.001, odds ratio = 3.2 (95% CI 2.84–3.53)) (Fig. 5D). To exclude the confounding influence of assembled contig lengths, we built a logistic regression with a binomial distribution to explore the association. It turned out that even after adjusting for contig length, significantly more VGs were detected in plasmids (odds ratio = 1.88 (95% CI 1.74–2.04), *p* < 0.001).

### The abundance of co-localized ARGs associates with infant gut microbial maturity

High ARG abundance is linked to low gut microbial maturity in infants [6], and the co-localization of ARGs may promote the persistence of this state in the infant’s gut. To shed light on this phenomenon, we explored the association between the abundance of co-localized ARGs and gut microbial maturity at 1 year of age.

**Fig. 6:**
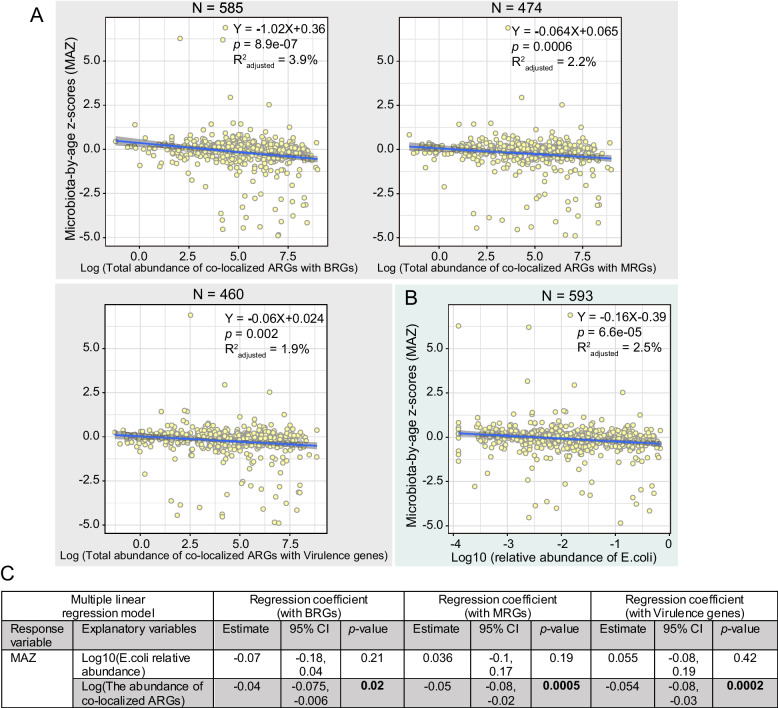
The association between the abundance of co-localized ARGs and infant gut microbial maturity. The association between log-transformed total abundance of co-localized ARGs (**A**) or *E. coli* relative abundance (**B**) and MAZ score. Confidence interval for the slope of the linear regression line is shown. The formula of the regression line, *p* value for slope, and adjusted R^2^ value are listed in the figures. The number of samples used in the linear regression analysis is denoted by N. **C** Multiple regression analysis evaluating whether *E. coli* is the sole determinant of the association between co-localized ARGs and infant gut microbial maturity

Linear regression analysis revealed that a higher abundance of co-localized ARGs was significantly correlated with lower MAZ scores (Fig. 6A), i.e., immaturity. However, given the vital role played by *E. coli* in providing co-localized ARGs to the microbial community, it is possible that the association between co-localized ARG abundance and gut maturity may depend solely on the abundance of *E. coli*. To investigate this hypothesis, we first fit a linear regression model with the log-transformed relative abundance of *E. coli* as the explanatory variable and MAZ score as the dependent variable and found an association between the two (Fig. 6B, estimate − 0.16 SD per log10 fold change, 95% CI [− 0.23, − 0.08], *p <* 0.001). This suggested that the high abundance of *E. coli* in the gut of 1-year-old infants was associated with low gut microbial maturity, which can also be verified by a gradual decline in *E. coli* relative abundance with age (Additional file 1: Figure S9). When we added the abundance of co-localized ARGs and BRGs to the model (Fig. 6C), we found that this was also associated with the MAZ score (*p =* 0.02) and, in fact, the significance and effect size between *E. coli* and MAZ changed (*p =* 0.21). Likewise, when we added the abundance of co-localized ARGs and MRGs or VGs to the model, this was also associated with the MAZ scores (*p =* 0.0005, 0.0002) and changed the significance and effect size between *E. coli* and MAZ (*p=* 0.19, 0.42) (Fig. 6C). Thus, after accounting for the abundance of co-localization in these cases, the abundance of *E. coli* was no longer a significant factor in the models. This suggests that *E. coli* is not a sole variable in terms of the association with maturity.

## Discussion

In this study, we comprehensively analyzed genetic co-localization involving ARGs and the association of this phenomenon with environmental factors and gut microbial maturation in a cohort of 662 Danish children. Collectively, the multidrug resistances in the infant gut microbiome were primarily associated with the broad-spectrum antibiotics fluoroquinolone and tetracycline and derived largely from *E. coli* (with an average relative abundance of 5.41%, the second most abundant species, Additional file 1: Figure S10). Multidrug resistance against fluoroquinolones and tetracycline has also been reported to be prevalent in ESBL producers (e.g., *E. coli*) isolated from multiple ecological settings, including water bodies and the feces of animals and healthy humans [46–48]. This pattern is consistent with the extensive use of these antibiotics in animal husbandry and medicine in recent decades and also involves the HGT of resistance genes on MGEs [49–54]. Besides, the bacterial diversity in the infant gut was low while *E. coli* was one of the major early colonizers with a high relative abundance (Additional file 1: Figure S9).

With the extensive use of other antimicrobial agents, co-selection of resistance to antibiotics, biocides, and/or metals has been commonly detected in bacteria from the environment, animal farming, and the human microbiome [10, 12, 54–58]. Similarly, we observed many ARGs and BRGs or MRGs co-occurring on the same contigs in the infant gut at frequencies significantly higher than those expected by chance, suggesting an enrichment effect. However, the extent to which antimicrobial agents and MGEs drive this enrichment effect, i.e., the mechanism of co-selection and the underlying drivers, is not entirely clear [59]. The statistical model showed that this enrichment was primarily found in Proteobacteria, especially in the clinically relevant bacterial family *Enterobacteriaceae*, independent of the high ARG content and length of contigs. The high prevalence of co-localization in the family *Enterobacteriaceae* was also observed in previous research [10, 12].

The presence of VGs together with ARGs may result in the emergence of novel “superbugs” [60]—pathogenic bacteria with multi-resistant phenotypes. A recent large genomics investigation confirmed abundant co-localization between VGs and ARGs in human pathogenic bacteria, and this co-occurrence or correlation was observed across various ecological settings [61–64]. Similarly, our study identified abundant co-localization in opportunistic pathogens such as *E. coli*, *H. parainfluenzae*, *Klebsiella spp.*, *Enterococcus* spp., and *Citrobacter* spp. in infants. Through the HGT of plasmids, multiple co-localized resistance genes can easily spread among bacteria. In the plasmids examined in this study, class 1 integrons were identified as the predominant genetic loci for the transfer of ARGs with BRGs and/or MRGs in the infant gut. This is consistent with previous work reporting that most known ARG cassettes are located in class 1 integrons [65], which have been identified in more than 70 clinical bacterial species and are affected by human activities [66]. Over the last few decades, QACs have been widely used as cationic surfactants in household products, and this has been accompanied by a concomitant increase in QAC resistance genes in bacteria, thereby increasing the risk of co-selection of antibiotic-resistant bacteria [67–69]. Accordingly, our study reveals that co-selection of QAC resistance genes and ARGs has occurred in healthy infants.

We explored mobile ARGs and VGs in the infant’s gut. Our results demonstrate that antibiotic treatment caused a short-term change in the total abundance of mobile ARGs in the infant gut, suggesting that antibiotic use facilitated the acute spread of resistance among bacteria, with MGEs as vectors. A similar association between oral antibiotic use and increased intestinal MGE abundance was reported in a study on the gut microbiome of fish [38]. Here, we found a significantly higher proportion of mobile VGs than mobile ARGs. The transfer of VGs is one of the main ways in which bacterial pathogens acquire virulence in the course of evolution [17], and, as reported here, a variety of VGs, including adherence factors, secretion systems, and toxins, have been detected in plasmids [70–72]. HGT events involving VGs pose a particular threat to health through, for example, the emergence of novel pathogens [73] and the formation of defense islands or pathogenicity islands over time [74].

The abundance of co-localized ARGs significantly influenced the maturation of the infant gut microbiome, with *E. coli* playing a vital role. As one of the earliest colonizers, the abundant resistance and VGs in *E. coli* (Additional file 1: Figure S11, S3) can grant it a substantial selective advantage. However, the prolonged persistence of *E. coli* may disrupt the subsequent colonization of beneficial commensal bacteria, thereby leading to a delay in the maturation of the infant gut microbiome. Early intervention is therefore essential. However, due to the high genetic variability of *E. coli* [75], its persistence and harmfulness to the host may vary considerably among strains [76, 77]. Future research focused on differentiating the effects of different *E. coli* strains on gut microbial maturation would be useful in identifying the strains for which intervention is most critical.

## Conclusion

In conclusion, we observed that co-localization between ARGs and other resistance and virulence genes was common in the early gut and was associated with gut bacteria that are indicative of low maturation. The most widespread form of co-localization involved tetracycline and fluoroquinolone resistance genes together with other ARGs. We evaluated the stochasticity of co-localization occurrence using enrichment scores for the first time. Co-localization of different resistance genes as well as virulence genes was most prevalent in Proteobacteria, as verified by statistical models excluding ARG content and contig length. Class 1 integrons were identified as the predominant genetic elements for co-localization of ARGs in the plasmids in the infant gut. We found that antibiotic administration caused a 45-day increase in the abundance of mobile ARGs, while non-mobile ARGs were unaffected. In addition, we found that a high abundance of virulence genes was associated with low maturation of the gut microbiome and observed that such genes showed an even higher potential for mobility than ARGs. Our study provides new insights into the maintenance and transmission of ARGs in the infant gut and indirectly emphasizes the need to apply caution in the use of antimicrobial agents in clinical practice, animal husbandry, and daily life to mitigate the escalation of resistance.

### Supplementary Information

**Additional file 1:** **Figure S1.** Emergence and co-selection of multidrug resistance in chromosomes or plasmids. A) Progressive acquisition of multiple resistances from mutations or gene transfer as a result of exposure to antimicrobial agents. Co-resistance represents the co-localization of multiple resistance genes in the same gene fragment. Cross-resistance represents a gene that confers resistance to multiple antimicrobials. B) Co-selection between multiple resistance genes upon exposure to any of the corresponding antimicrobials. This figure is adapted from the work of Cantón and Ruiz-Garbajosa [8]. **Figure S2.** Summary of antibiotic usage for 662 infants during the first year. **Figure S3.** Distribution of gene richness and abundance among bacterial species carrying (A) BRGs, (B) MRGs, (C) virulence genes, and (D) MGEs in the infant gut.**Figure S4.** Total abundance (sum of GCPM) of (A) virulence genes in log scale, (B) BRGs in log scale, (C) MRGs, and (D) MGEs in log scale in the infant gut. The labels on the x-axis describe (A) virulence factors, (B) biocides, (C) metals, and (D) categories of MGEs. The red dot in the scatter plot represents the median value of the total abundance.**Figure S5.** Co-localization bubble chart representing the drug classes related to 42 ABRGs. The size of the bubble is proportional to the abundance of the ABRGs. The drug classes in red represent those used in this cohort. **Figure S6.** The pecent of varience explained by different phyla in the co-localization of ARGs and BRGs/MRGs. **Figure S7.** Distribution and Co-localization of ARGs, BRGs/VGs, and MGEs on plasmids or Chromosome in the infant gut. A) The respective abundance (percentage) of contigs, resistance genes, VGs, and mobile genetic elements in chromosomes and plasmids. B) Venn diagram depicting the number of contigs with resistance genes and MGEs in chromosomes and plasmids. **Figure S8.** The representative co-localization contigs carrying the different types of integrons on plasmids. Based on the ARGs on the 22 representative contigs, 16 representative contigs carrying class 1 integrons were chosen and listed in the figure (each representative contig represents a group consisting of 1 to 16 contigs). The complete integron in each contig is highlighted in gray. For ease of viewing, we only marked the approximate location of the attC recombination site, the constitutive promoter Pc for the gene cassettes, the promoter for the integrase gene PintI, and the integration site attI. To target potential mobile ARGs, we only focused on the co-localization contigs carrying MGEs on the plasmids. The location and length of genes are proportional to the actual conditions. **Figure S9.** The mean relative abundance of E. coli in the gut of infants at one week, one month, one year, four years, five years, and six years of age based on 16S sequencing data. **Figure S10.** The percent of the 45 most abundant bacterial species in the infant gut. **Figure S11.** E. coli drives the bimodal distribution of BRGs, MRGs, and virulence genes in the infant gut. A) Density plot of gene richness among infants and Spearman correlation analyses between the richness of ARGs and other genes. Y-axis (not shown in the density plot) represents the number of infants. The linear regression curve and confidence interval are plotted as an illustration; inference was performed with the Spearman correlation coefficient R and the corresponding p-value (*p* < 0.05 as the significance cutoff). B) Average silhouette width associated with PAM clustering when different numbers of clusters were used, and the total abundance and richness of genes in the two clusters. A high average silhouette value indicates strong clustering. k = 2 was the optimal number of clusters. C) Importance of the top five bacterial species to the grouping of PAM clusters, as determined with a Random Forest-based approach using the mean decrease in accuracy and the relative abundance of E. coli in the two clusters. Species are ordered top-to-bottom as most-to-least important.

**Additional file 2:** **Table S1.** Mobile virulence genes.

## Data Availability

All sequencing data are available in the Sequence Read Archive (SRA) under the accession number PRJNA715601. The R code and source data for data analysis have been uploaded to http://mibi.galaxy.bio.ku.dk/R_script_Coselection/.
