# Supplementary material for: Co-localization of antibiotic resistance genes is widespread in the infant gut microbiome and associates with an immature gut microbial composition
Source: Microbiome. 2024 May 10;12:87. doi: 10.1186/s40168-024-01800-5 (PMC11084089; doi:10.1186/s40168-024-01800-5)
Supplement: Supplementary file 2 — Additional file 1: Figure S1. Emergence and co-selection of multidrug resistance in chromosomes or plasmids. A) Progressive acquisition of multiple resistances from mutations or gene transfer as a result of exposure to antimicrobial agents. Co-resistance represents the co-localization of multiple resistance genes in the same gene fragment. Cross-resistance represents a gene that confers resistance to multiple antimicrobials. B) Co-selection between multiple resistance genes upon exposure to any of the corresponding antimicrobials. This figure is adapted from the work of Cantón and Ruiz-Garbajosa [8]. Figure S2. Summary of antibiotic usage for 662 infants during the first year. Figure S3. Distribution of gene richness and abundance among bacterial species carrying (A) BRGs, (B) MRGs, (C) virulence genes, and (D) MGEs in the infant gut.Figure S4. Total abundance (sum of GCPM) of (A) virulence genes in log scale, (B) BRGs in log scale, (C) MRGs, and (D) MGEs in log scale in the infant gut. The labels on the x-axis describe (A) virulence factors, (B) biocides, (C) metals, and (D) categories of MGEs. The red dot in the scatter plot represents the median value of the total abundance.Figure S5. Co-localization bubble chart representing the drug classes related to 42 ABRGs. The size of the bubble is proportional to the abundance of the ABRGs. The drug classes in red represent those used in this cohort. Figure S6. The pecent of varience explained by different phyla in the co-localization of ARGs and BRGs/MRGs. Figure S7. Distribution and Co-localization of ARGs, BRGs/VGs, and MGEs on plasmids or Chromosome in the infant gut. A) The respective abundance (percentage) of contigs, resistance genes, VGs, and mobile genetic elements in chromosomes and plasmids. B) Venn diagram depicting the number of contigs with resistance genes and MGEs in chromosomes and plasmids. Figure S8. The representative co-localization contigs carrying the different types of integrons on plasmids. Based [file 40168_2024_1800_MOESM1_ESM.pdf]

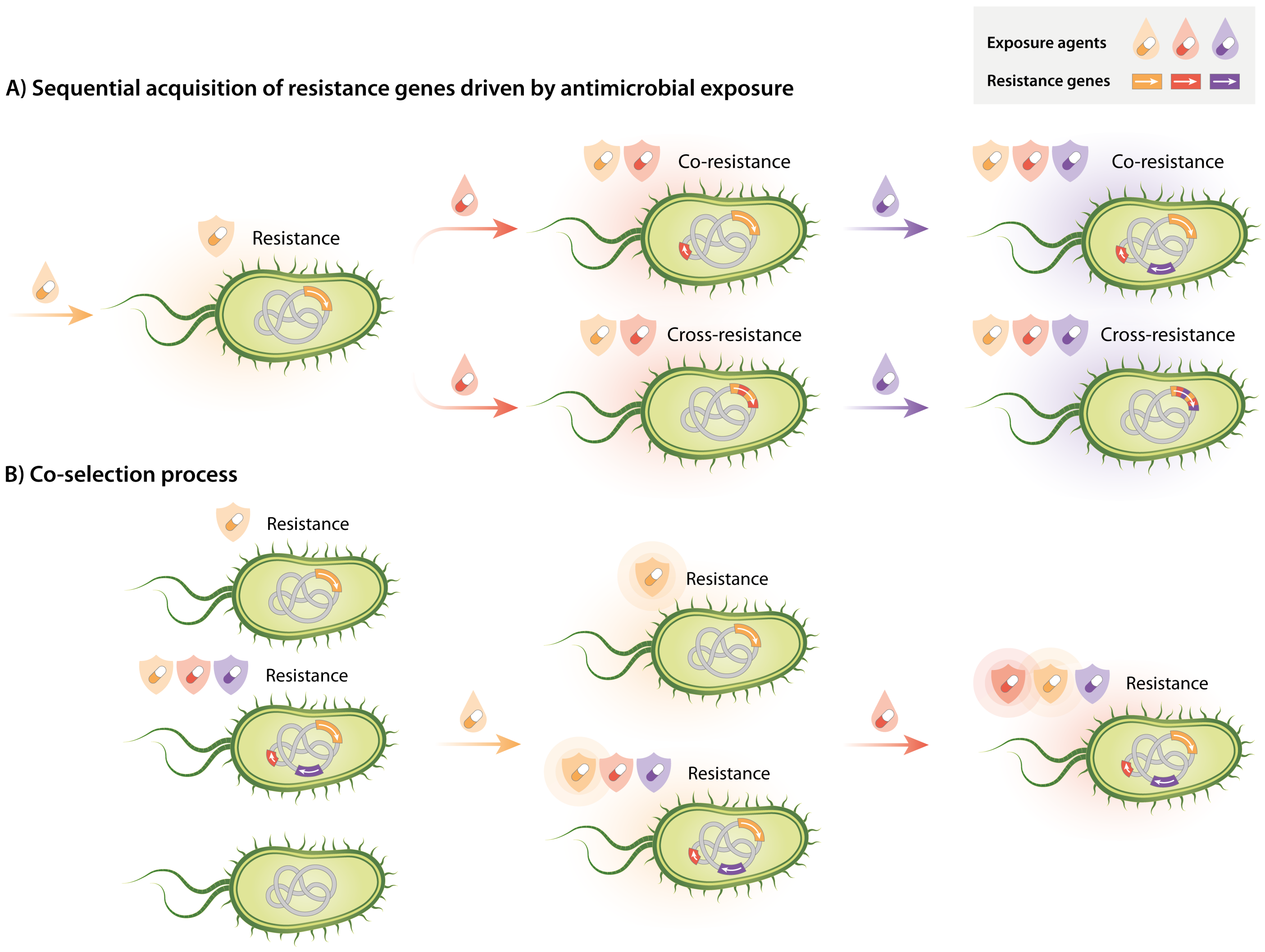

Fig S1. Emergence and co-selection of multidrug resistance in chromosomes or plasmids. A) Progressive acquisition of multiple resistances from mutations or gene transfer as a result of exposure to antimicrobial agents. Co-resistance represents the co-localization of multiple resistance genes in the same gene fragment. Cross-resistance represents a gene that confers resistance to multiple antimicrobials.
B) Co-selection between multiple resistance genes upon exposure to any of the corresponding antimicrobials. This figure is adapted from the work of Cantón and Ruiz-Garbajosa [78].

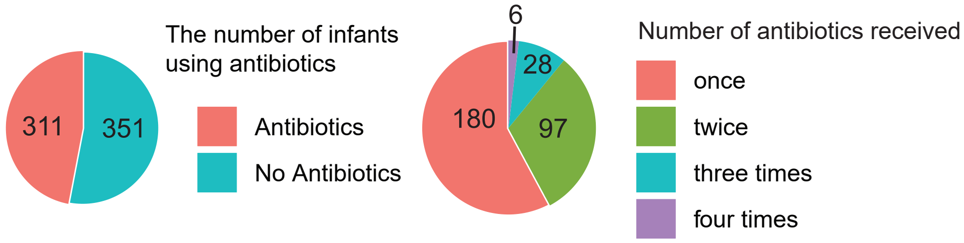

Fig S2. Summary of antibiotic usage for 662 infants during the first year.

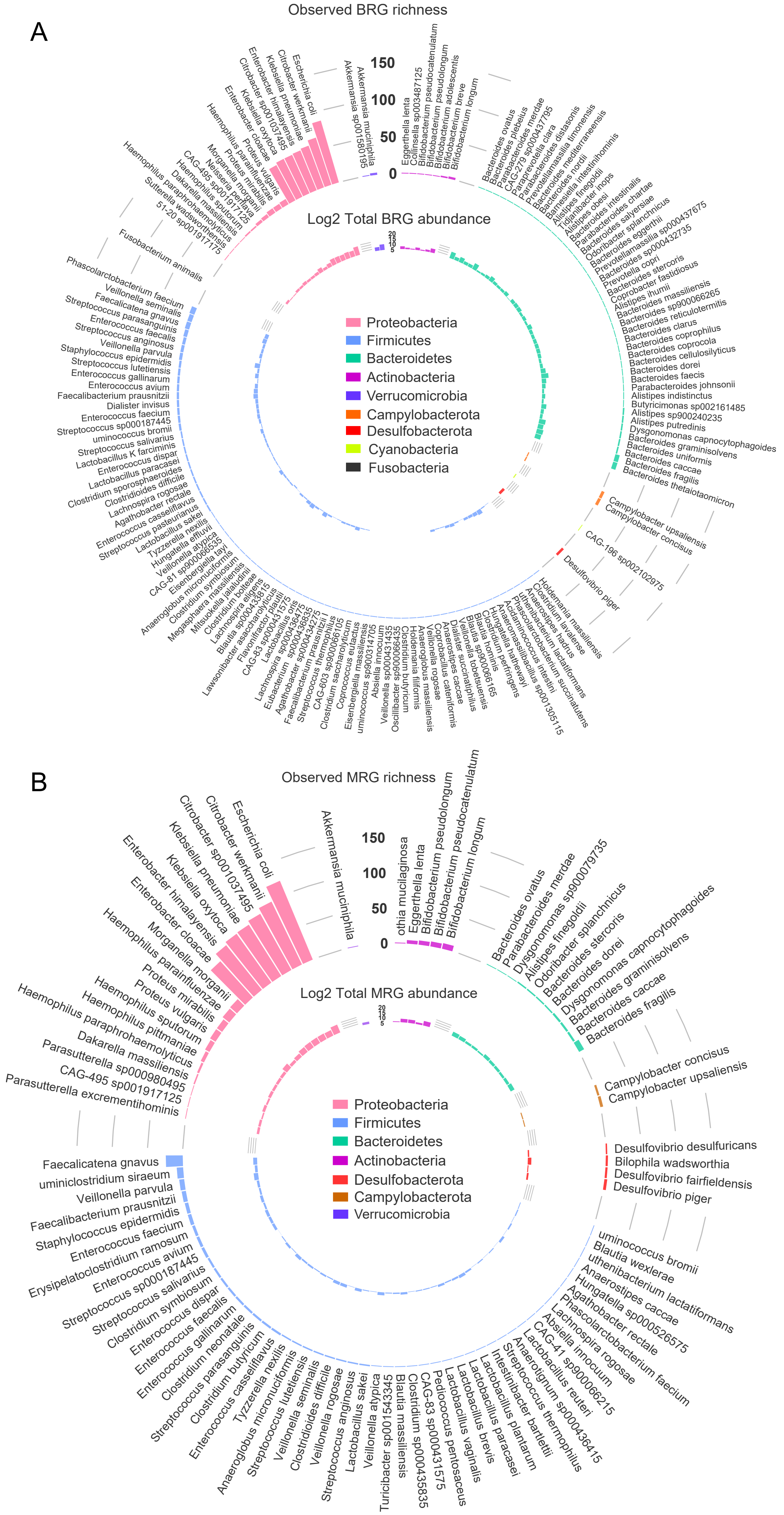

Fig S3. Distribution of gene richness and abundance among bacterial species carrying (A) BRGs, (B) MRGs, (C) virulence genes, and (D) MGEs in the infant gut.

C

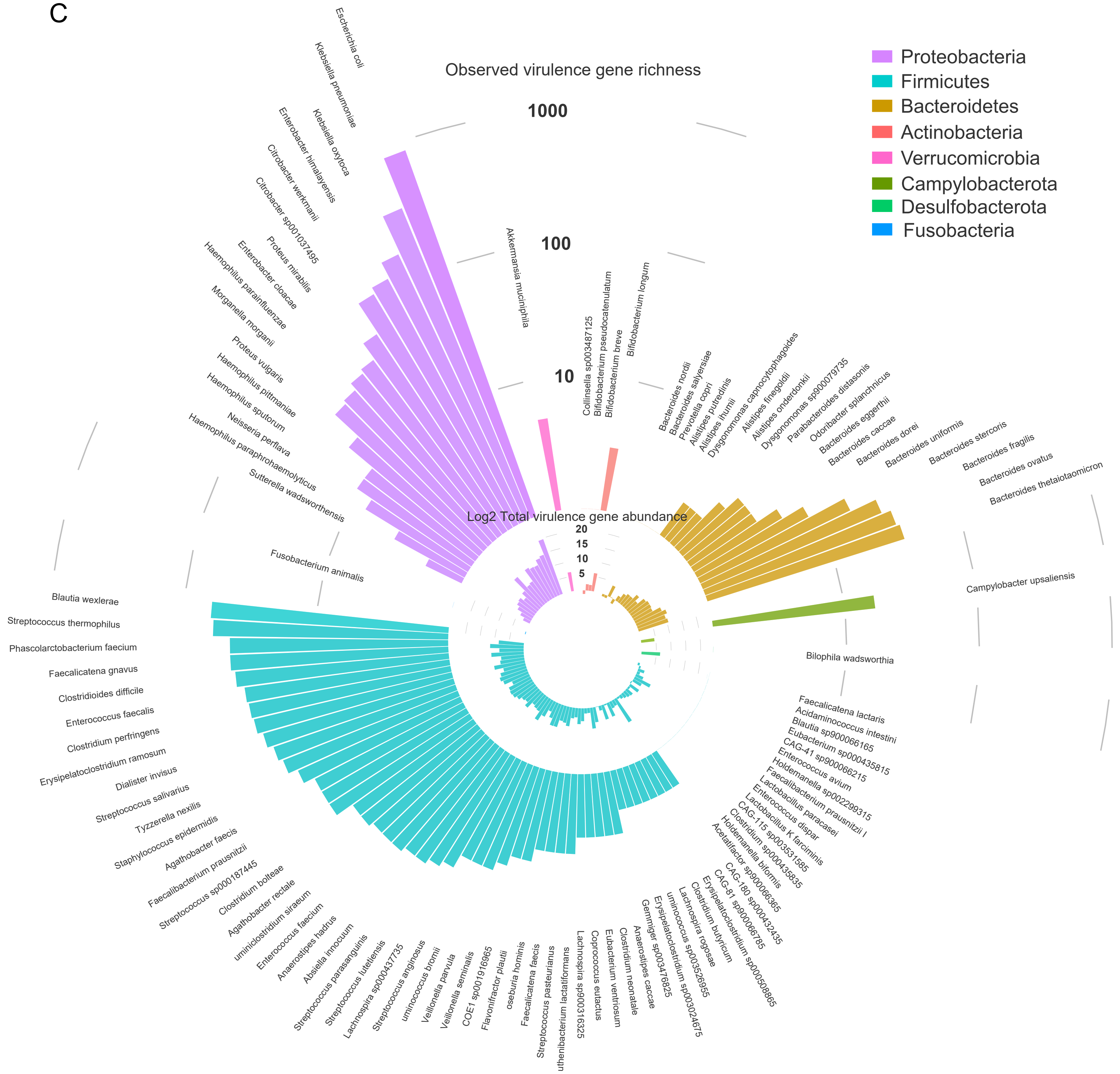
Observed virulence gene richness
1000
100
10
Log2 Total virulence gene abundance
20
15
10
5
Proteobacteria
Firmicutes
Bacteroidetes
Actinobacteria
Verrucomicrobia
Campylobacterota
Desulfobacterota
Fusobacteria
Escherichia coli
Klebsiella pneumoniae
Klebsiella oxytoca
Enterobacter himalayensis
Citrobacter werkmanii
Citrobacter sp001037495
Proteus mirabilis
Enterobacter cloacae
Haemophilus parainfluenzae
Morganella morganii
Proteus vulgaris
Haemophilus pittmaniae
Haemophilus sputorum
Neisseria perflava
Haemophilus paraphrohaemolyticus
Sutterella wadsworthensis
Fusobacterium animalis
Akkermansia muciniphila
Collinsella sp003487125
Bifidobacterium pseudocatenulatum
Bifidobacterium breve
Bifidobacterium longum
Bacteroides nordii
Bacteroides salyersiae
Prevotella copri
Alistipes putredinis
Alistipes ihumii
Dysgonomonas capnocytophagoides
Alistipes finegoldii
Alistipes onderdonkii
Dysgonomonas sp900079735
Parabacteroides distasonis
Odoribacter splanchnicus
Bacteroides eggerthii
Bacteroides caccae
Bacteroides dorei
Bacteroides uniformis
Bacteroides stercoris
Bacteroides fragilis
Bacteroides ovatus
Bacteroides thetaiotaomicron
Campylobacter upsaliensis
Bilophila wadsworthia
Faecalicatena lactaris
Acidaminococcus intestini
Blautia sp900066165
Eubacterium sp000435815
CAG-41 sp900066215
Enterococcus avium
Holdemanella sp002299315
Faecalibacterium prausnitzii I
Lactobacillus paracasei
Enterococcus dispar
Lactobacillus K farciminis
CAG-115 sp003531585
Clostridium sp000435835
Holdemanella biformis
Acetatifactor sp900066365
CAG-180 sp000432435
CAG-81 sp900066785
Erysipelatoclostridium sp000508865
Clostridium butyricum
Lachnospira rogosae
uminococcus sp003526955
Erysipelatoclostridium sp003024675
Gemmiger sp003476825
Anaerostipes caccae
Clostridium neonatale
Eubacterium ventriosum
Coprococcus eutactus
Lachnospira sp900316325
uthenibacterium lactatiformans
Streptococcus pasteurianus
Faecalicatena faecis
oseburia hominis
Flavonifractor plautii
COE1 sp001916965
Veillonella seminalis
Veillonella parvula
uminococcus bromii
Streptococcus anginosus
Lachnospira sp000437735
Streptococcus lutetiensis
Streptococcus parasanguinis
Absiella innocuum
Anaerostipes hadrus
Enterococcus faecium
uminiclostridium siraeum
Agathobacter rectale
Clostridium bolteae
Streptococcus sp000187445
Faecalibacterium prausnitzii
Agathobacter faecis
Staphylococcus epidermidis
Tyzzerella nexilis
Streptococcus salivarius
Dialister invisus
Erysipelatoclostridium ramosum
Clostridium perfringens
Enterococcus faecalis
Clostridioides difficile
Faecalicatena gnavus
Phascolarctobacterium faecium
Streptococcus thermophilus
Blautia wexlerae

D

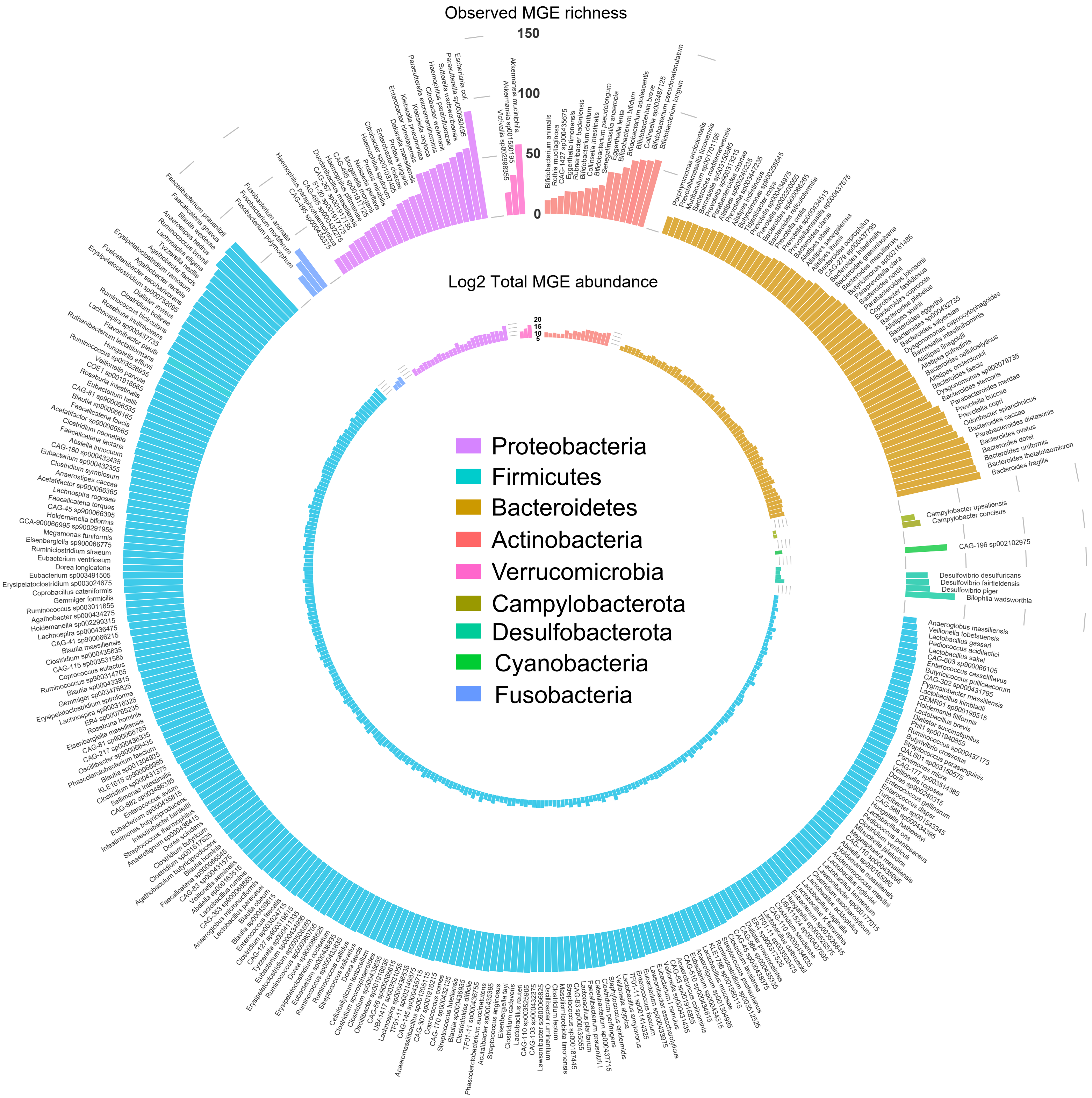
Observed MGE richness
150
100
50
0
Log2 Total MGE abundance
20
15
10
5
Proteobacteria
Firmicutes
Bacteroidetes
Actinobacteria
Verrucomicrobia
Campylobacterota
Desulfobacterota
Cyanobacteria
Fusobacteria

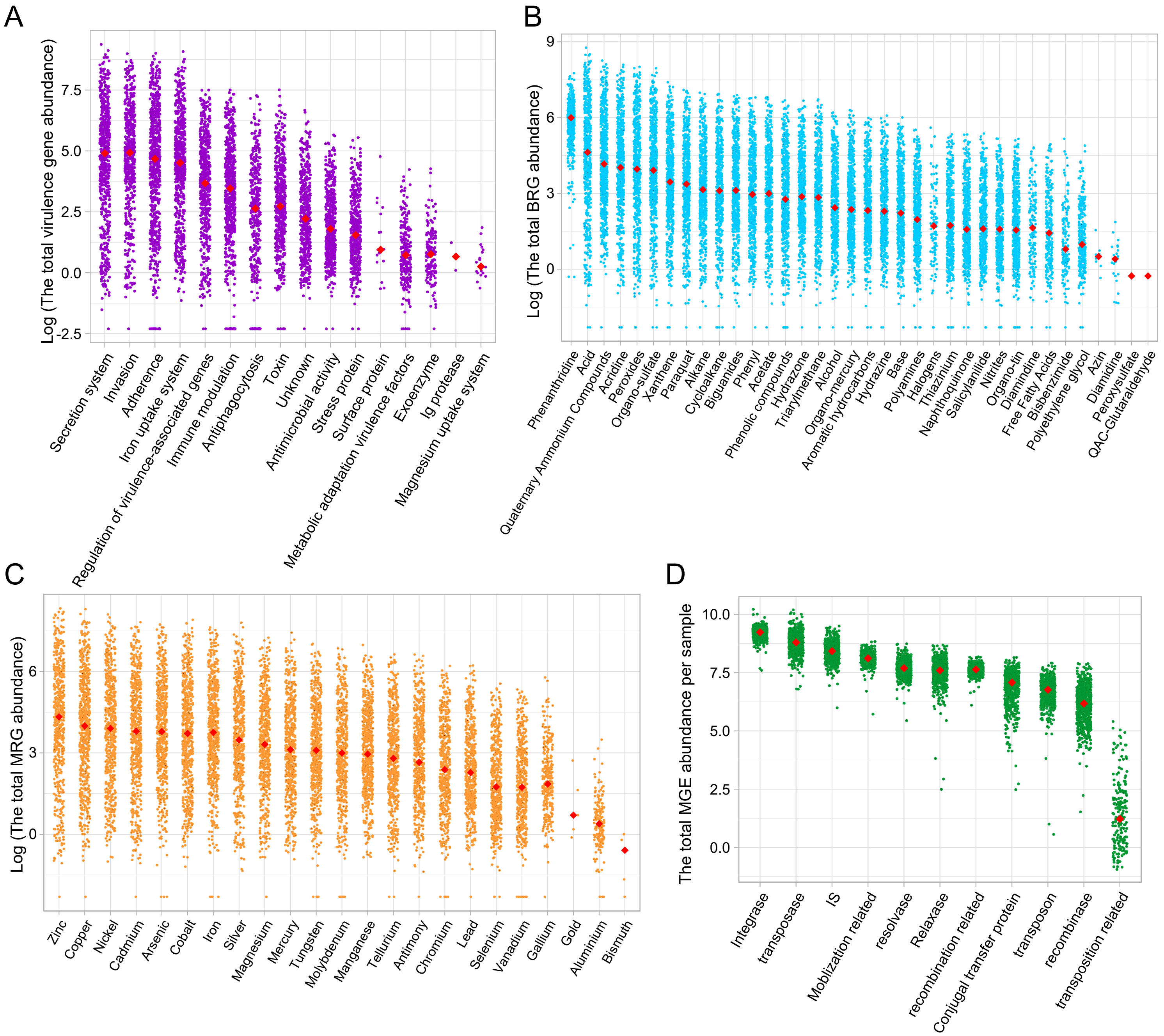

Fig S4. Total abundance (sum of GCPM) of (A) virulence genes in log scale, (B) BRGs in log scale, (C) MRGs, and (D) MGEs in log scale in the infant gut. The labels on the x-axis describe (A) virulence factors, (B) biocides, (C) metals, and (D) categories of MGEs. The red dot in the scatter plot represents the median value of the total abundance.

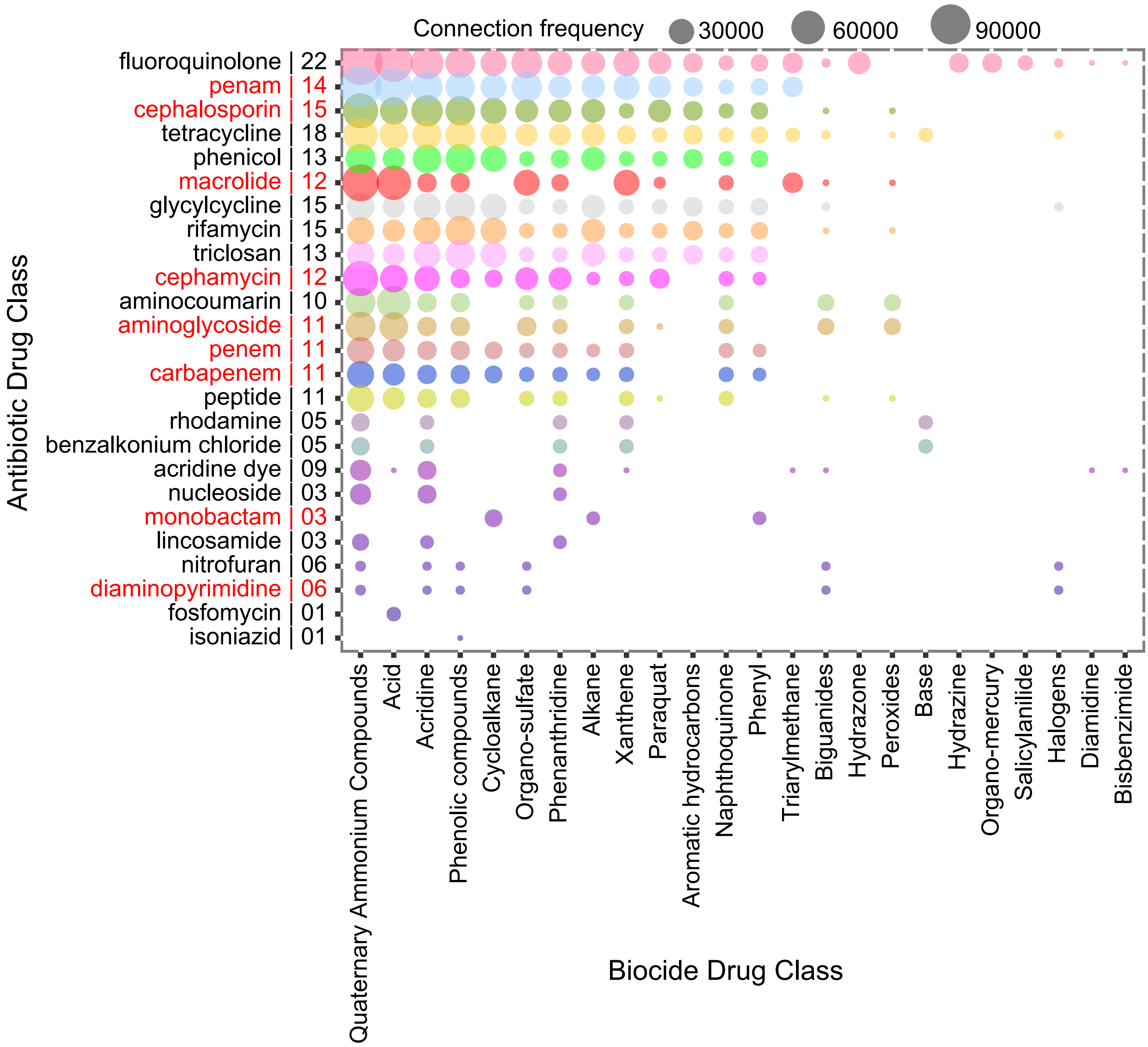

Fig S5. Co-localization bubble chart representing the drug classes related to 42 ABRGs. The size of the bubble is proportional to the abundance of the ABRGs. The drug classes in red represent those used in this cohort.

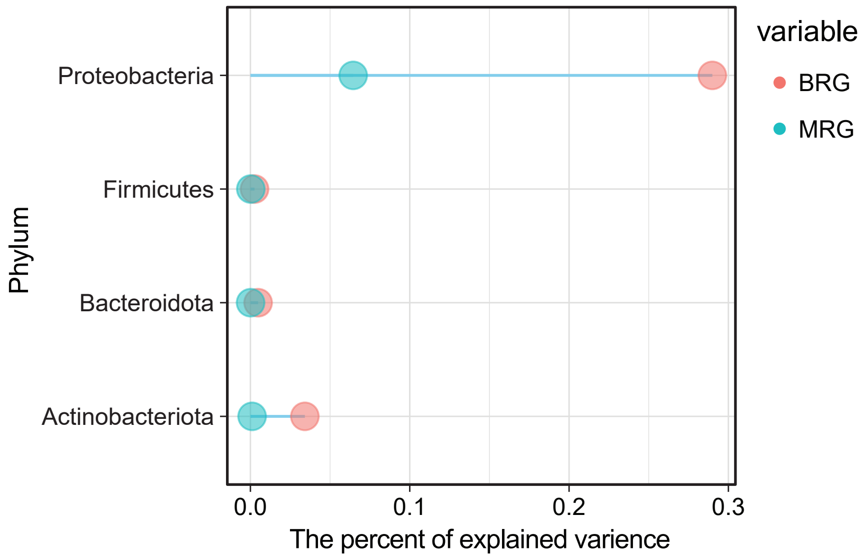

Fig S6. The pecent of varience explained by different phyla in the co-localization of ARGs and BRGs/MRGs

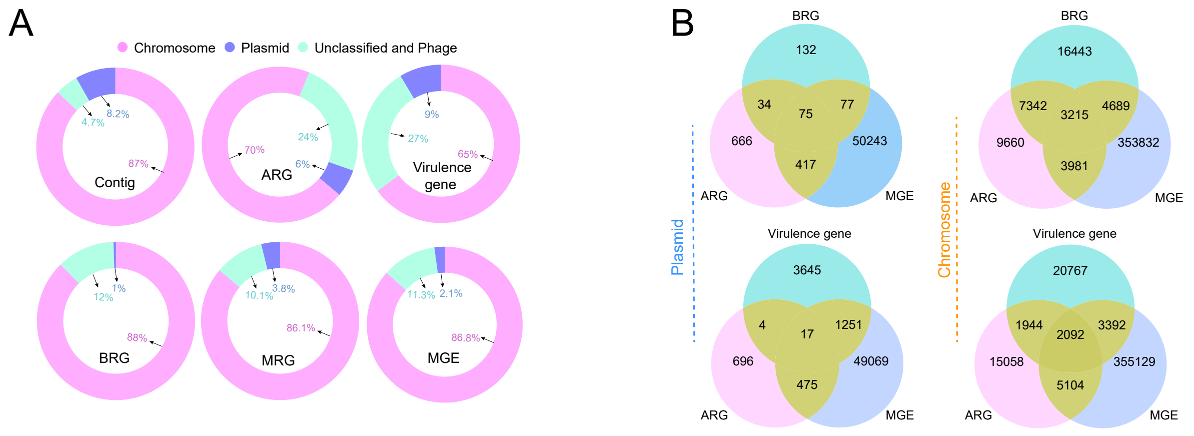

Fig S7. Distribution and Co-localization of ARGs, BRGs/VGs, and MGEs on plasmids or Chromosome in the infant gut. A) The respective abundance (percentage) of contigs, resistance genes, VGs, and mobile genetic elements in chromosomes and plasmids. B) Venn diagram depicting the number of contigs with resistance genes and MGEs in chromosomes and plasmids.

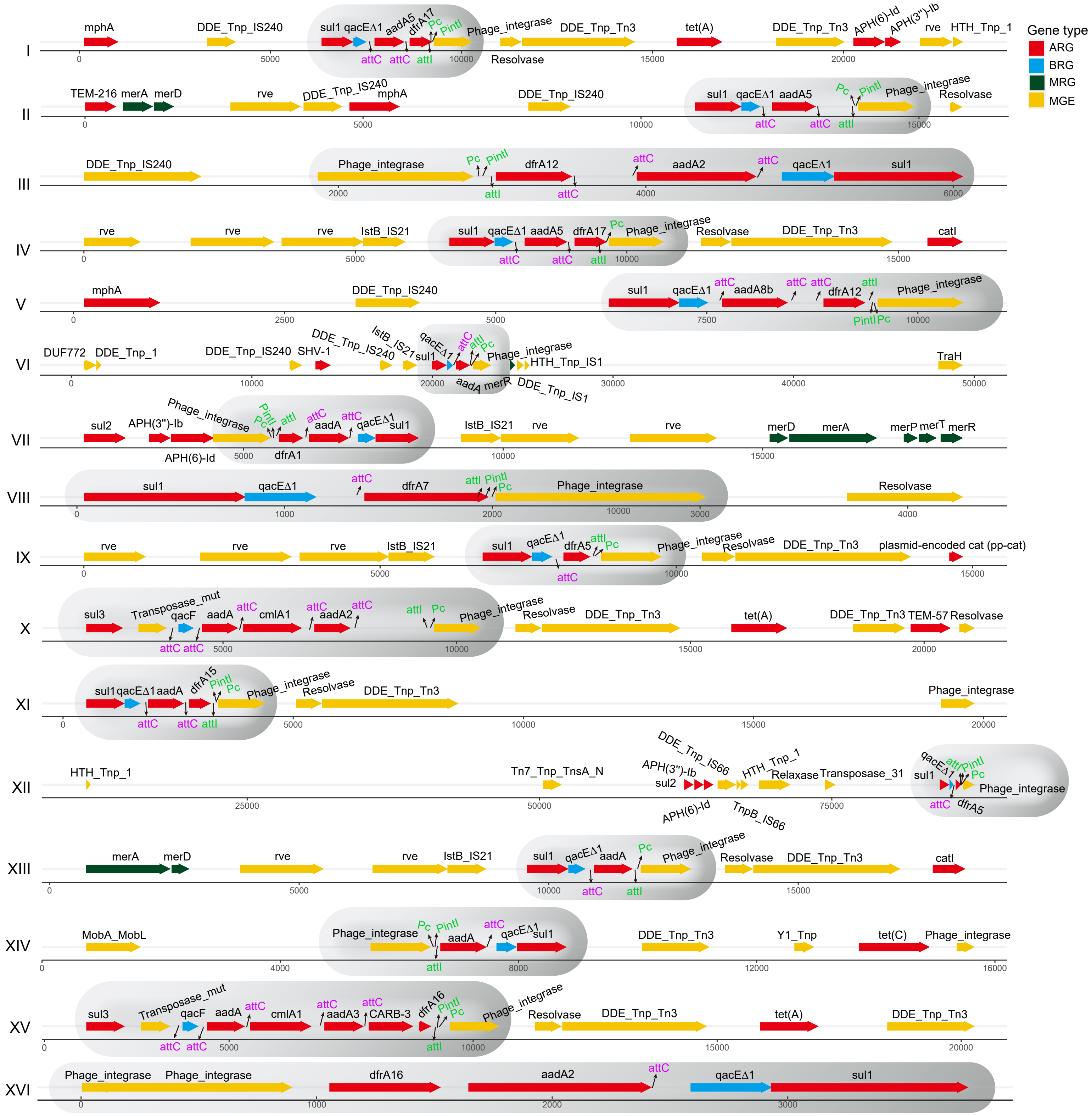

Fig S8. The representative co-localization contigs carrying the different types of integrons on plasmids. Based on the ARGs on the 22 representative contigs, 16 representative contigs carrying class 1 integrons were chosen and listed in the figure (each representative contig represents a group consisting of 1 to 16 contigs). The complete integron in each contig is highlighted in gray. For ease of viewing, we only marked the approximate location of the attC recombination site, the constitutive promoter Pc for the gene cassettes, the promoter for the integrase gene PintI, and the integration site attI. To target potential mobile ARGs, we only focused on the co-localization contigs carrying MGEs on the plasmids. The location and length of genes are proportional to the actual conditions.

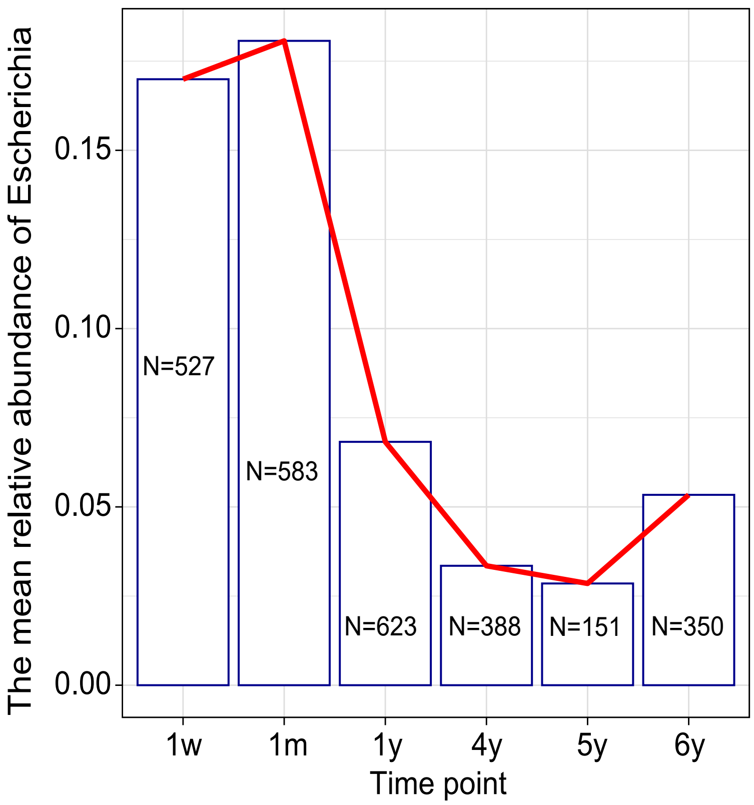

Fig S9. The mean relative abundance of E. coli in the gut of infants at one week, one month, one year, four years, five years, and six years of age based on 16S sequencing data.

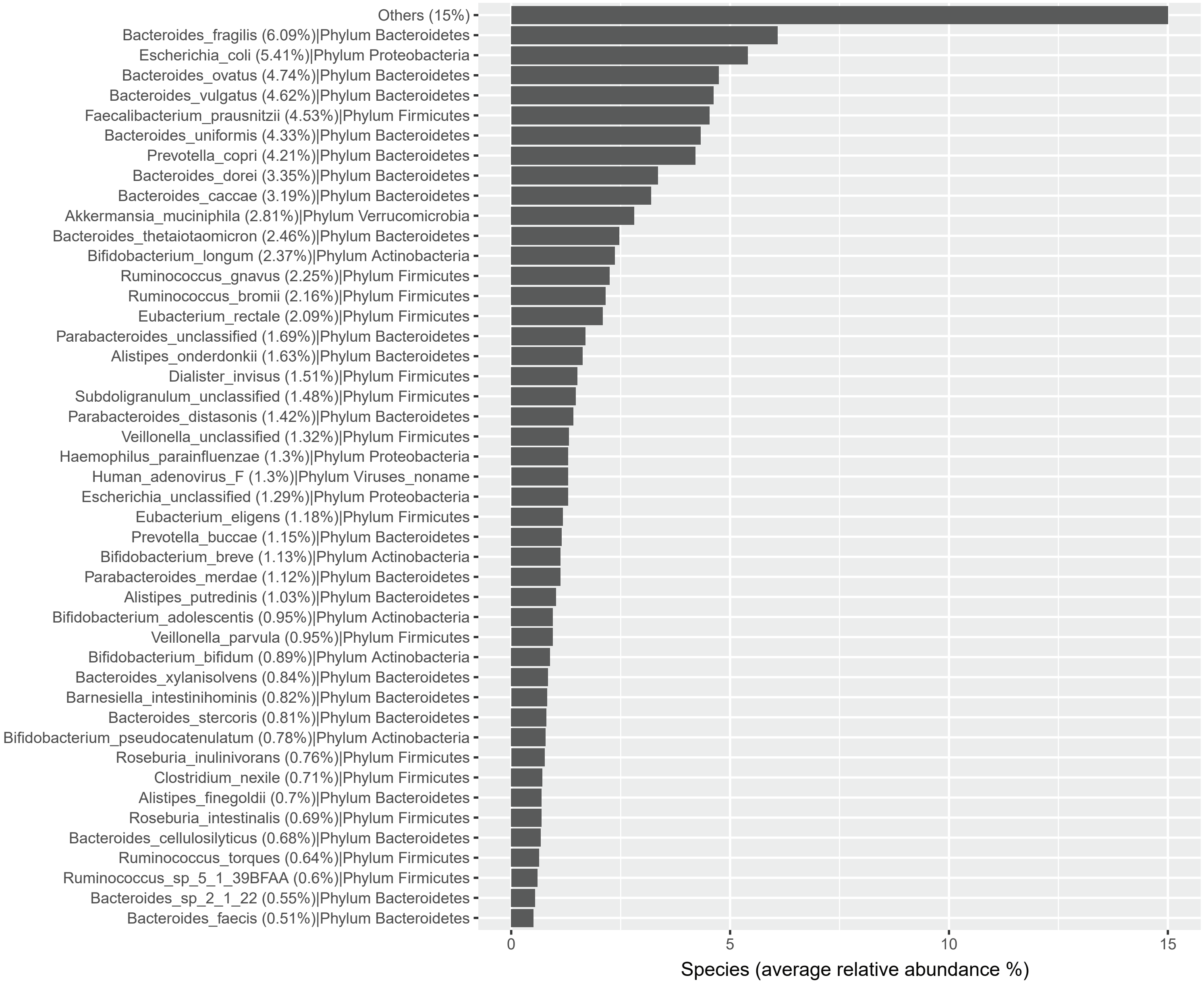

Fig S10. The percent of the 45 most abundant bacterial species in the infant gut.

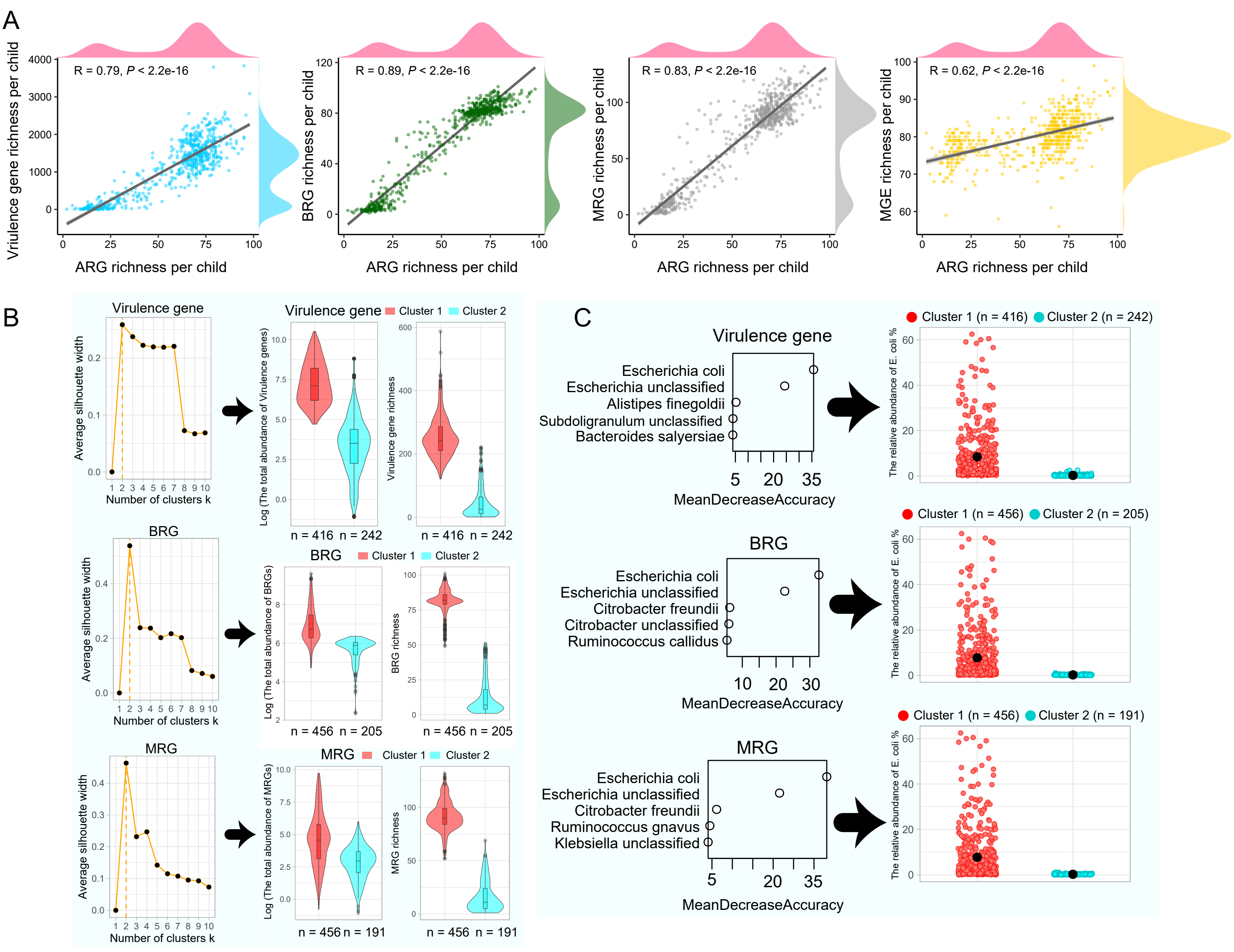

Fig S11. E. coli drives the bimodal distribution of BRGs, MRGs, and virulence genes in the infant gut.
A) Density plot of gene richness among infants and Spearman correlation analyses between the richness of ARGs and other genes. Y-axis (not shown in the density plot) represents the number of infants. The linear regression curve and confidence interval are plotted as an illustration; inference was performed with the Spearman correlation coefficient R and the corresponding p-value ($p < 0.05$ as the significance cutoff).
B) Average silhouette width associated with PAM clustering when different numbers of clusters were used, and the total abundance and richness of genes in the two clusters. A high average silhouette value indicates strong clustering. k = 2 was the optimal number of clusters. **C)** Importance of the top five bacterial species to the grouping of PAM clusters, as determined with a Random Forest-based approach using the mean decrease in accuracy and the relative abundance of E. coli in the two clusters. Species are ordered top-to-bottom as most-to-least important.
